# Uncovering the Mechanisms Responsible for Why Language Learning May Promote Healthy Cognitive Aging

**DOI:** 10.3389/fpsyg.2017.02217

**Published:** 2017-12-15

**Authors:** Mark Antoniou, Sarah M. Wright

**Affiliations:** The MARCS Institute for Brain, Behaviour and Development, Western Sydney University, Sydney, NSW, Australia

**Keywords:** bilingualism, language learning, cognitive aging, healthy aging, language typology

## Abstract

One of the great challenges facing humankind in the 21st century is preserving healthy brain function in our aging population. Individuals over 60 are the fastest growing age group in the world, and by 2050, it is estimated that the number of people over the age of 60 will triple. The typical aging process involves cognitive decline related to brain atrophy, especially in frontal brain areas and regions that subserve declarative memory, loss of synaptic connections, and the emergence of neuropathological symptoms associated with dementia. The disease-state of this age-related cognitive decline is Alzheimer’s disease and other dementias, which may cause older adults to lose their independence and rely on others to live safely, burdening family members and health care systems in the process. However, there are two lines of research that offer hope to those seeking to promote healthy cognitive aging. First, it has been observed that lifestyle variables such as cognitive leisure activities can moderate the risk of Alzheimer’s disease, which has led to the development of plasticity-based interventions for older adults designed to protect against the adverse effects of cognitive decline. Second, there is evidence that lifelong bilingualism acts as a safeguard in preserving healthy brain function, possibly delaying the incidence of dementia by several years. In previous work, we have suggested that foreign language learning programs aimed at older populations are an optimal solution for building cognitive reserve because language learning engages an extensive brain network that is known to overlap with the regions negatively affected by the aging process. Here, we will outline potential future lines of research that may uncover the mechanism responsible for the emergence of language learning related brain advantages, such as language typology, bi- vs. multi-lingualism, age of acquisition, and the elements that are likely to result in the largest gains.

## Introduction

One of the great challenges facing humankind in the twenty-first century is dealing with the problems associated with an aging population. Over-60-year-olds are the fastest growing age group on earth. By 2050, the number of people over 60 is set to triple, eclipsing 2 billion worldwide (Department of Economic and Social Affairs Population Division, 2007). As the number of older adults increases, so too will the demands and costs associated with an aging population, placing increasing pressure on families, health systems, economies, and governments. The typical aging process is characterized by age-related decline in a number of cognitive subsystems (Park et al., 2002; Drachman, 2006). Certain brain structures are particularly affected by the aging process, such as frontal areas, the hippocampus, and the entorhinal cortex (Gómez-Isla et al., 1996; MacPherson et al., 2002; Bertoni-Freddari et al., 2003). Reduced function may be observed in working memory, declarative memory, as well as the interaction between declarative and procedural memory (Harrington and Haaland, 1992; Grady and Craik, 2000). The disease state of cognitive decline is Alzheimer’s disease (and other dementias), characterized by a gradual progressive difficulty with learning and retaining new information. Pharmacological trials have had little success in slowing down the progression of Alzheimer’s disease (Salloway et al., 2014). This has led to increasing calls to treat the disease proactively using behavioral stimulations, ideally before symptoms manifest (Selkoe, 2012).

Two promising lines of research have developed in parallel that offer some hope to combating age-related cognitive decline. On the one hand are studies demonstrating that environmental enrichment may result in positive brain changes. Studies of animals reared in standard vs. enriched enclosures have demonstrated the effects of environmental enrichment on the brain, namely denser dendritic connections resulting from stimulation (Volkmar and Greenough, 1972; Greenough et al., 1985). Such findings concerning environmental enrichment have been mirrored in investigations of lifestyle variables associated with healthy brain aging in humans: education, physical and mental stimulation, occupation, and leisure activities have all been linked to positive outcomes in cognitive aging (Kramer et al., 2004; Staff et al., 2004; Valenzuela and Sachdev, 2006; McDowell et al., 2007; Brayne et al., 2010; Foubert-Samier et al., 2012). These observations have led to the development of numerous plasticity-based interventions that aim to use environmental enrichment proactively by prescribing cognitively stimulating training regimens such as crossword puzzles (Verghese et al., 2003), math exercises (Kawashima et al., 2005), brain training (Ball et al., 2002), and computer-based interventions (Smith et al., 2009), and these cognitive improvements have been shown to persist over time (Mahncke, 2006). The resulting improvements have been observed in healthy adults, and encouragingly, also in those with mild cognitive impairment (Belleville et al., 2011), and even in those diagnosed with Alzheimer’s disease (Bottino et al., 2005).

In parallel to the development and emergence of the cognitive training literature, evidence has been accumulating concerning the aging-related benefits of bilingualism. It was once thought that use of multiple languages (bilingualism) led to cognitive impairments (Goodenough, 1926). However, carefully conducted scientific studies later showed it to result in cognitive improvements (Peal and Lambert, 1962), an outcome since reinforced by a further 30 years of research. The evidence now suggests that experience with two languages confers a general ‘bilingual advantage,’ with improvement in executive function (Bialystok et al., 2004), metalinguistic awareness (Cummins, 1978), cognitive flexibility, creative thinking, and perhaps even several years’ delay in the onset of dementia (Bialystok et al., 2007). Multilingualism is a better predictor of cognitive ability than age, age at immigration, education, or sex (Kavé et al., 2008). These cognitive advantages that have been associated with bilingualism have neural correlates. For example, bilinguals demonstrate greater white matter integrity in old age compared to monolingual speakers (Luk et al., 2011). It has been suggested that this results in enhanced structural and functional connectivity that provides the neural basis for cognitive reserve. Bilingual older adults also show less steep cognitive decline than those who only speak one language (Bialystok, 2009). However, in recent years, the robustness of a bilingual advantage has been hotly debated—questioned by some who have failed to replicate it (Duñabeitia and Carreiras, 2015; Paap et al., 2015), but staunchly defended by its proponents (Bialystok et al., 2016). This debate itself highlights the absence of a detailed and systematic understanding of the factors that would underlie such an advantage. Interestingly, certain research laboratories consistently observe data patterns supporting a bilingual advantage, while other laboratories consistently find no advantage. It has even been suggested that bilingual advantages may reflect publication bias (de Bruin et al., 2015); but both the significant and non-significant findings are so systematic that it is much more likely that other factors (e.g., linguistic, experiential) are involved (Bialystok et al., 2015).

In the sections that follow, we will review the bilingual cognitive aging literature with a view to exploring the potential mechanisms responsible for the emergence of language learning related brain advantages and how these may be investigated prospectively in longitudinal language learning studies in adult learners.

## Bilingualism and Executive Function

Many studies have reported that bilingualism yields advantages in executive function. Gold et al. (2013) found that bilingual older adults showed better task-switching performance than monolinguals in a color-shape task where participants categorize images by their color (blue or red) and shape (square and circle). Furthermore, fMRI scans taken during this task revealed decreased activation in the bilinguals’ left lateral frontal cortex and cingulate cortex, an indication of more efficient executive functioning, when compared to a monolingual control group, and this difference was consistent across both the younger and older participants. Additionally, bilinguals outperformed monolinguals in episodic memory recall and letter fluency, but not the categorical fluency task (Ljungberg et al., 2013). Learners of French as a second language outperformed monolinguals on a grammaticality judgment task (ignoring conflict introduced through misleading semantic content) and a non-verbal visual search task (Janus et al., 2016). Older adult bilinguals, including those who acquired their second language in adulthood, exhibited improved cognitive function (general intelligence and reading) compared to monolinguals (Bak et al., 2014). When exposed to a non-verbal switching task, monolinguals showed activation in the right inferior frontal cortex and the anterior cingulate whereas bilinguals showed activation in the left inferior frontal cortex and left striatum, both areas that underlie language control (Garbin et al., 2010). Older adult bilinguals processed distracting information more efficiently than their monolingual peers when completing the Flanker task (Ong et al., 2017). Aging bilinguals may also show less steep declines in executive function as they progress from healthy aging to mild cognitive impairment to probable Alzheimer’s disease (Anderson et al., 2017). Collectively, these studies suggest that bilinguals show an advantage for non-linguistic cognitive abilities, particularly executive functions.

However, in recent years, a growing number of studies have raised questions regarding the robustness (and in some cases, the validity) of these bilingual advantage claims. This has led to attempts to understand the factors that give rise to the bilingual advantage in cognitive function and also explain its absence under certain conditions. Bilinguals’ executive control abilities may be enhanced due to higher processing demands, and it has been argued that this may build cognitive reserve in the elderly (Costa and Sebastián-Gallés, 2014). Although the exact mechanisms are not agreed upon, it is generally thought that bilinguals’ cognitive and brain reserves share the same mechanism as executive control processing (Grant et al., 2014). The complexity of the underlying cognitive processes may play a crucial role, with greater inhibitory demands resulting in greater benefit (Valian, 2015a,b). If correct, bilingualism may ultimately delay clinical Alzheimer’s disease symptoms by protecting brain regions that subserve executive control (frontostriatal and frontoparietal) rather than those that subserve memory (medial temporal lobe) *per se* (Gold, 2015). Further, the potentially mediating effect of age of second language acquisition on executive functions is not well understood, and thus neither is its potential impact on the structure of the brain (Duñabeitia and Carreiras, 2015; Paap et al., 2015). Recently, Bialystok (2017) put forth an experience-dependent plasticity framework to evaluate the brain and cognitive modifications attributed to bilingualism. It was concluded that research broadly supports a relation between bilingualism and cognitive brain outcomes in infants and children, younger and older adults, and patients, however, behavioral studies with young adults, commonly fail to show these effects. This interpretation is consistent with findings in the executive function literature. Executive functions reach their peak in young adulthood (Park et al., 2002), and thus greater variability in executive functions, as measured by behavioral tasks, are more likely to be observed in older adulthood (when cognitive functions decline; Bialystok et al., 2008) or in childhood (when the foundations of cognitive processing are being established; Bayliss et al., 2003). Thus, it seems reasonable that bilingual advantages would be easier to detect either in early or later life.

## Bilingualism and Cognitive Reserve

Cognitive reserve refers to the brain’s resilience to neuropathological damage, resulting from experience-based neural changes associated with a physically and mentally stimulating lifestyle (Whalley et al., 2004). Stern (2012) proposes two possible mechanisms for cognitive reserve: neural reserve, according to which differences in the resilience of already established networks, and neural compensation, according to which some individuals are better able to compensate for brain decline by using alternative networks. Evidence exists for both possibilities, and thus, the mechanisms responsible for cognitive reserve are a matter of ongoing research.

It is perhaps then unsurprising that the mechanism via which bilingualism improves the brain’s resistance to neuropathology is not understood. Recent scholarly work has uncovered several potentially fruitful avenues concerning how bilingualism might build cognitive reserve focusing on the interactions between cognitive reserve and variables known to affect bilingualism (Calvo et al., 2016), as well as the brain networks that subserve memory (Grant et al., 2014), brain metabolic connectivity (Perani et al., 2017), and the presence of Alzheimer’s disease biomarkers in cerebrospinal fluid (Estanga et al., 2017). Nevertheless, numerous studies present evidence suggesting that bilingualism results in brain changes in healthy subjects. Higher degrees of bilingualism have been linked to better lexical memory performance (Jafari et al., 2015). Bilinguals have higher white matter integrity than monolinguals in the corpus callosum extending to the superior and inferior longitudinal fasciculi, and also stronger anterior to posterior functional connectivity (Luk et al., 2011). Aging bilinguals outperformed monolinguals on the Flanker task, and had increased gray matter in the anterior cingulate cortex, whereas monolinguals showed decreased gray matter in the dorsolateral prefrontal cortex (Abutalebi et al., 2015b). Further, brain regions that support executive control significantly overlap with brain regions recruited for language control (Abutalebi and Green, 2016). The brain plasticity effects of lifelong bilingualism are thought to contribute to cognitive reserve and delay the onset of symptoms associated with dementia (Guzmán-Vélez and Tranel, 2015; Perani and Abutalebi, 2015). There is also evidence that bilingual brains are better able to accommodate anatomical and physiological brain changes and deterioration without exhibiting the expected increase in behavioral symptoms. Bilingual patients with Alzheimer’s disease exhibited greater amounts of brain atrophy than monolingual patients (radial width of the temporal horn and the temporal horn ratio; Schweizer et al., 2012). Bilingual patients also showed substantially greater impairment of glucose uptake in frontotemporal and parietal regions (Brodmann areas 9, 47, 40, and 21) and in the left cerebellum relative to monolingual patients (Kowoll et al., 2016). This evidence supports the view that lifelong bilingualism may benefit the brain by making use of efficient or alternative neural networks in the event of age-related decline and that greater amounts of brain atrophy are required before the disease manifests, which may possibly delay the incidence of dementia.

## Does Bilingualism Protect Against Dementia?

The evidence for a protective effect of bilingualism on the incidence of dementia is considerable. Numerous studies have examined dementia incidence in hospital records and concluded that bilingualism exerts a protective effect. The first such study by Bialystok et al. (2007) revealed that lifelong bilinguals showed a delay in the onset of symptoms of dementia by 4 years compared to monolinguals. Similarly, Craik et al. (2010) reported that bilingual patients had been diagnosed with Alzheimer’s disease 4.3 years later and had reported the onset of symptoms 5.1 years later than the monolingual patients. Additionally, Woumans et al. (2015) found that bilingual patients had been diagnosed with Alzheimer’s disease 4.8 years later and presented symptoms 4.6 years later than monolingual patients. Similarly, speakers of two or more languages had a delayed onset of Alzheimer’s disease by up to 5 years and a protective effect was significant when speaking at least two to four languages (Freedman et al., 2014). Looking at specific dementia subtypes, bilingualism delayed the age at onset in the behavioral but not in the aphasic variants of Frontotemporal Dementia (Alladi et al., 2017), a finding consistent with the observation that bilingualism has positive effects on behavioral syndromes but not on language disorders. Indeed the effects of bilingualism on language functions are not always beneficial (e.g., smaller vocabulary size in a single language, slower lexical processing, reduced verbal fluency etc.). Further, a similar study by Alladi et al. (2016) comparing monolingual and bilingual stroke patients found that bilinguals had a significantly lower frequency of post-stroke dementia and mild cognitive impairment but the same frequency of post-stroke aphasia. Moreover, Atkinson (2016) reviewed nine papers and concluded that frequent use of two languages over a lifetime may be protective against dementia, and that inconsistencies arise due to study design or definitions of bilingualism. This evidence supports the protective effect of bilingualism against the symptoms of dementia (Bialystok et al., 2016), as well as the later onset of symptoms of mild cognitive impairment compared to monolinguals (Bialystok et al., 2014). Bilingual individuals diagnosed with single-domain amnesic mild cognitive impairment demonstrated a later age of diagnosis than did monolinguals (Ossher et al., 2013). Cerebral hypometabolism was more severe in the left hemisphere in bilinguals with Alzheimer’s dementia compared to monolinguals, but nevertheless bilinguals outperformed monolinguals on memory tasks, suggesting that bilinguals are better able to compensate for the loss of brain structure and function (Perani et al., 2017). Furthermore, exposure to foreign language instruction during childhood and adolescence has been associated with lower risk of developing mild cognitive impairment in old age (Wilson et al., 2015). Bilingualism has been associated with delayed onset of dementia and is also observed in illiterate patients (Alladi et al., 2013). Taken together, this body of work suggests that bilingual experience delays the onset of neurodegenerative disease.

However, an increasing number of studies have failed to detect a bilingual advantage in dementia incidence. A cohort design with non-immigrant samples found no significant differences in the onset of dementia between mono- and bilingual subjects (Lawton et al., 2015). No significant association was found between non-native English speakers and the incidence of dementia or Alzheimer’s disease (Sanders et al., 2012). In that study, non-native English speakers with at least 16 years of education had a fourfold *increased risk* for dementia compared to those with less education, which is an unusual finding and inconsistent with past literature on the protective effect of education. Yeung et al. (2014) found no association between dementia diagnoses for bilinguals (English as a second language and bilingual English) and monolinguals. Zahodne et al. (2014) reported that adult learners of English had better memory and executive function than monolinguals, but that bilingualism was not associated with cognitive decline or dementia. Fuller-Thomson (2015) has claimed that the support for a bilingual advantage in dementia onset is questionable, and has attributed the current state of the literature to the file drawer problem, a bias against publishing non-significant findings from small studies with low to medium statistical power, a selection bias due to use of patients from a memory clinic, potential recall bias in caregivers’ reporting of age of onset of dementia and confounding by immigration status. Indeed, Clare et al. (2016) did not observe any advantage for delay in Alzheimer’s onset in Welsh-English bilinguals over English monolinguals (but see Bak, 2016 for a discussion of how this finding is conflated by the unusual situation of monolingual migration). A recent meta-analysis concluded that bilingualism offers no protection against cognitive decline (Mukadam et al., 2017), and that retrospective studies supporting the bilingual protective effect against dementia are marred by methodological confounds. Note, however, that this meta-analysis has already been criticized as misleading and incomplete (Woumans et al., 2017). In sum, these studies have led to questions regarding the robustness (or in some cases the validity) of the bilingual dementia advantage.

In order to resolve the debate, attempts have been made to understand the role of any potential mediating factors and experimental confounds. Gollan et al. (2011) claim that higher degrees of bilingualism are associated with increasingly later age of diagnosis and symptom onset, but this may be obscured by interactions between education and bilingualism, and a failure to obtain objective measures of bilingualism. Bak and Alladi (2014) highlight that although there exists support that bilingualism has a positive effect on cognition throughout the lifespan, common misconceptions concerning the nature of bilingualism persist, including that bilingualism is an unusual phenomenon, the holistic nature of bilingualism and its effects on cognition and bilingual diversity. Further, Fuller-Thomson’s (2015) and Lawton et al.’s (2015) assertions that monolinguals and bilinguals do not differ in the onset of dementia have been criticized as overly simplistic. Bak and Alladi (2016) point out that it is necessary to study the effects of bilingualism separately from those of immigration and education, and to use data from both community-based approaches and memory clinics. Bak (2016) further highlights the importance of addressing confounding variables in bilingualism, aging and dementia research which include heterogeneity, migration, social factors, differences in general intelligence and the related issue of reverse causality.

The above literature review has demonstrated that bilingualism yields executive functioning advantages, and these may contribute to building cognitive reserve, which may ultimately delay the onset of dementia. The exact mechanisms are not agreed upon, and there exists counterevidence that limits the generalisability of these claims. A possible fruitful avenue is the recent suggestion that sustained activation of noradrenergic signaling pathways associated with bilingualism could provide a possible mechanism linking current and previous results supporting a delayed onset of dementia in bilinguals (Bak and Robertson, 2017). The following sections of this article are devoted to proposing additional possible explanations and mechanisms that may provide parsimonious explanations for the seemingly conflicting findings currently in the literature.

## Age of Acquisition

The majority of studies examining a bilingual advantage in cognitive aging have considered the effects of lifelong experience on cognitive function and decline. Consequently, very little attention has been paid to the age of acquisition of the second language. Age of acquisition of the second language positively correlates with cortical thickness in the left inferior frontal gyrus and a thinner cortex in the right inferior frontal gyrus (Klein et al., 2014). Encouragingly, there is evidence of a positive effect of language experience on individuals who acquired their languages later in life. Both early and late bilinguals were found to have more efficient executive networks than monolinguals. Proficient late bilinguals showed the greatest advantage in conflict resolution, whereas early bilinguals showed enhanced monitoring processes (Tao et al., 2011). Interestingly, Abutalebi et al. (2015a) found that age of acquisition did not correlate with gray matter volumes in the left or right inferior parietal lobules in aging bilinguals.

Age of acquisition is a complex variable in that it not only represents the level of input experienced by a learner, where early age of acquisition results in more years of exposure, but also potentially differing patterns of language use between speakers who acquired their second language in early or in later life. Such differences in language use may modulate the cognitive advantages associated with bilingualism. For instance, balanced bilinguals showed age-related decline in their inhibition abilities (as indexed by the Simon task), whereas dominant bilinguals showed no evidence of age-related decline (Goral et al., 2015). Further, when looking purely at amount of input, age of acquisition may need to be evaluated differently in older adulthood than it is for younger adults. For example, Tao et al. (2011) define early acquisition as occurring by an average of 4.0 years, and late acquisition as occurring by an average of 12.3 years. Although this difference in years of second language input might be marked for young adults, it is possible that this difference is negligible for those over the age of 65. Additionally, age of acquisition may result in executive control differences, not because of biological or maturational constraints on language learning, but because age of acquisition may be a proxy for a set of environmental differences that are necessarily associated with early vs. late second language learning (Tao et al., 2011). Indeed, those learning a language later due to migration will necessarily use their languages differently than someone learning a heritage language at an early age. Future longitudinal language training studies are needed to determine how age of acquisition modulates any cognitive improvements resulting from language learning, and whether it truly is never too late to begin language learning.

## Neuroimaging Studies of Language Learning in Adults

A large neuroscientific literature has demonstrated that lifelong bilingualism alters the structure of the brain. Recent work has confirmed that brain changes may also be observed in healthy adults following relatively short periods of language training, and a picture is emerging concerning the brain changes that subserve dynamic uses of language (see **Table 1** for a summary of these findings). Interpreters who learned a foreign language intensively for 3 months showed increases in hippocampus volume and in cortical thickness in the left middle frontal gyrus, inferior frontal gyrus, and superior temporal gyrus, relative to controls; those with high proficiency showed structural malleability (right hippocampus and the left superior temporal gyrus) and struggling interpreters presented larger gray matter increases in the middle frontal gyrus (Mårtensson et al., 2012). Foreign language training in students increased white matter including pathways in the right hemisphere, and correlated with gain in second language ability not observed in controls (Hosoda et al., 2013). English natives who spent 5 months learning Swiss German showed structural changes in the left inferior frontal gyrus which correlated with increased second language proficiency (Stein et al., 2012). Moreover, structural changes in gray matter (inferior parietal cortex and left inferior frontal gyrus) and white matter (anterior corpus callosum) have been repeatedly linked to second language proficiency (Stein et al., 2014). Successful learners of a tonal language showed significant differences in language-related regions in the brain and a more coherent, integrated multi-path brain network compared to less successful learners, whereas monolinguals relied on different brain networks to process tonal and lexical information (Yang et al., 2015).

**Table 1:** Summary of empirical studies investigating brain changes in healthy adults due to language training.

Study	Sample	Training type	Training duration	Results
Hosoda et al., 2013	**24 language learners:** Japanese speaking students in basic level English courses (*M*_age_ = 20.1). **20 controls:** Japanese speaking students in basic level English courses. Age matched. Not participating in e-learning.	Computer-based e-learning.	60 idioms/words per week including spelling, meaning and pronunciation for 16 weeks.	Gray matter volume increased in right IFGop. White matter increased in right sub IFGop. Increased connectivity between IFGop caudate head pathway and dorsal pathway in RH.
Mårtensson et al., 2012	**14 conscript interpreters:** (*M*_age_ = 19.9), learning Arabic (*n* = 4), Dari (*n* = 8), or Russian (*n* = 2). No prior knowledge of these languages. **17 controls:** Medical and cognitive science students (*M*_age_ = 20.6).	Face-to-face classes and individual language studies.	Participants learned the language to fluency in 10 months. Scans conducted prior to start and after 3 months training. Participants learned 300–500 new words per week, and studied daily.	Increased cortical thickness in LH for dorsal MFG, IFG, STG, and right MFG and IFG. Greater hippocampal volume increase for interpreters, with proficiency related to right hippocampal volume and thickness of left STG. Changes in MIFG cortical thickness positively related to teacher ratings of student effort.
Stein et al., 2012	**10 English-speaking exchange students:** Learning German in Switzerland (*M*_age_ = 17.5).	Immersion and face-to-face classes.	5 months of second language learning including intensive 3-week course.	German proficiency related to increase in gray matter density for left IFG and ATL.
Yang et al., 2015	**21 English-speaking language learners:** No history of learning a tone language (*M*_age_ = 20.61). **13 passive controls:** No history of learning a tone language. Completed no language training (*M*_age_ = 20.8).	Computer-based.	Three 30 min training sessions per week for 6 weeks. Participants learned 48 Mandarin pseudoword picture pairs.	Increased activation of bilateral posterior MTG/AG for learners indicating that they treated tonal information as lexical. At completion of training, learners showed decreased activation for bilateral MEFG, MIFG, IFG, SMA, ACC, insula, and MTG relative to non-learners.

*ACC, anterior cingulate cortex; AG, angular gyrus; ATL, anterior temporal lobe; IFG, inferior frontal gyrus; IFGop, inferior frontal gyrus pars opercularis; LH, left hemisphere; MEFG, medial frontal gyrus; MIFG, middle frontal gyrus; MTG, middle temporal gyrus; RH, right hemisphere; SMA, supplementary motor area; STG, superior temporal gyrus.*

In sum, support has been found for second language experience-induced brain changes via increased gray matter density and white matter integrity in children, young adults, and the elderly; with such changes occurring rapidly following short-term language training. Further, these changes are sensitive to age, age of acquisition, proficiency or performance level, language-specific characteristics, and individual differences (Li et al., 2014).

## The Role of Language Typology

One factor that has so far hardly played a role in the bilingual advantage debate concerns the languages that bilinguals use. A powerful factor might be the match or mismatch in typology (types of language structure, e.g., where verbs occur, use of affixes, etc.). Such structural features indeed influence learning of a foreign language (Cenoz, 2003; Antoniou et al., 2015), so they may also affect the likelihood of language-related advantages emerging, though this has not yet been examined.

There are two possible, and contrasting, roles that language typology could play in determining whether cognitive advantages result from foreign language learning. First, typologically different languages might be more demanding to learn because they share few linguistic commonalities. It has recently been proposed that tasks that are more cognitively engaging will yield greater cognitive benefits (e.g., photographic training is superior to watching documentaries; Park et al., 2014), and for language learners, the benefits may be greatest when demands exceed their available cognitive resources (Schroeder and Marian, 2016). Second, typologically similar languages could lead to rapid learning because they share linguistic commonalities (and cognates), leading, after attainment of some proficiency, to competition between the languages exceeding that between distant languages (Weber and Cutler, 2004; Broersma and Cutler, 2011; Cutler, 2015). This in turn would require greater suppression, placing greater demands on the executive function system and its associated brain structures (prefrontal cortex; Stein et al., 2012, inferior parietal lobule; Mechelli et al., 2004, anterior cingulate; Abutalebi et al., 2012, basal ganglia; Zou et al., 2012, and putamen; Abutalebi et al., 2013). These alternatives predict opposite results regarding the appearance of cognitive benefit, but in both cases, the relationship between a learner’s native and target languages would influence the demands on their cognitive resources.

We refer to the first possibility as the *processing complexity effect*, according to which greater cognitive improvements will ensue from learning typologically differing languages, as these require more effort to learn and existing native-language knowledge cannot be relied on. The alternative is the *interference inhibition effect*, according to which greater cognitive improvements will ensue from learning typologically similar languages, because similar languages interfere more, increasing demands on executive control systems in the brain.

As noted above, studies to date have typically reported advantages for individuals using multiple languages for many years. But rigorous investigation of how language learning affects cognitive function must: (a) measure the cognitive abilities of interest *prior to*, as well as post, language learning, and (b) experimentally manipulate who learns what language. Neither can be done with individuals who are already bilingual. Systematic investigation of the effects outlined above will require longitudinal experiments in which participants will be cognitively assessed both prior to and after completing language training. This allows (1) control of extraneous variables (e.g., education, Gollan et al., 2011; socioeconomic status, Calvo and Bialystok, 2014; quality and quantity of language input, Sorace and Serratrice, 2009), (2) assignment of participants to target language, and (3) experimental manipulation of the relationship between the target language and the learner’s native language (i.e., typological similarity).

Although these possibilities have not yet been tested systematically, there is some evidence that typological distance will result in reliable brain differences. For example, Abutalebi et al. (2015a) observed greater gray matter volumes in the brains of aging Chinese bilinguals relative to monolinguals, specifically in the left and right inferior parietal lobules. Importantly, when comparing Cantonese-English and Cantonese-Mandarin bilinguals, both groups showed greater gray matter volumes for the right inferior parietal lobule, but only Cantonese-Mandarin bilinguals showed greater gray matter volumes for the left inferior parietal lobule. Although preliminary, this observation is consistent with our hypothesized interference inhibition effect, suggesting that two similar languages result in greater competition and place greater demands on the executive control system, requiring more inhibition to avoid language interference. This may result in brain differences that are more prominent for speakers of typologically similar languages. This interference inhibition effect could potentially go some way to providing a parsimonious explanation for conflicting findings in the literature.

## The Cognitive Benefits of Additional Language Learning: Bilinguals vs. Multilinguals

Experience with multiple languages is thought to yield cognitive advantages that promote healthy cognitive aging, although the mechanisms responsible are not fully understood. If our proposed interference inhibition effect is correct, then conditions that increase interference will yield the greatest benefits. Therefore, knowledge of a greater number of languages would likely increase competition and the required inhibitory control and thus yield a greater cognitive benefit. However, we note that presently it is not clear if experience with a greater number of languages results in an additive benefit. There is some evidence suggesting that this may indeed be the case. Aging bilinguals and multilinguals maintain higher levels of cognitive functioning than monolinguals, irrespective of immigration and education levels (Kavé et al., 2008). Chertkow et al. (2010) found evidence for a later age of onset of Alzheimer’s disease symptoms in multilinguals as compared with monolinguals, whereas a limited effect was found between bilinguals compared to monolinguals. Participants who practiced more than two languages presented a lower risk of cognitive impairment without dementia, compared to bilinguals. Progressing from two to three languages was associated with a sevenfold protection against cognitive impairment without dementia (Perquin et al., 2013). There is some evidence that this multilingual benefit is mediated by age. Older trilingual adults showed larger advantages on cognitive reserve than bilinguals. However, younger trilingual adults and children showed the same advantages as bilinguals on inhibitory control measures. Trilingual infants and toddlers performed worse than bilinguals on memory generalization tasks (Schroeder and Marian, 2016). In sum, it is not clear if multilingualism brings about greater cognitive benefits than bilingualism, although the present evidence suggests that it is likely to emerge under certain circumstances.

## Language Learning Studies With Older Adults

We have established that there is evidence to suggest that lifelong bilingualism may enhance executive functions, contribute to cognitive reserve, and possibly protect against Alzheimer’s disease. However, whether language learning initiated in older adulthood could yield cognitive improvements remains an open research question. Previous research has shown that both healthy older adults and those at risk of neural dysfunction have demonstrated positive brain changes to training (Valenzuela et al., 2003). This indicates that the benefits of mental stimulation are not limited to younger adults, but that even the aging brain retains its neuroplasticity, and thus training-related benefits may still be observed in older participants. Given that language learning engages an extensive neural network (Rodríguez-Fornells et al., 2009) that overlaps with the network affected by age-related cognitive decline (Raz, 2000), a tantalizing possibility is that language learning may promote healthy brain aging in older adults (see Antoniou et al., 2013 for a full review). Although there is currently very little research in this area, there are positive signs that this may well be the case (**Table 2**). For example, Bak et al. (2016) found language learning advantages for task switching using the elevator task with reversal. In this task, participants listen to a sequence of three different tones: low, mid, and high. Participants are required to count the mid tones, add one for the high tones, and subtract one for the low tones. The advantage demonstrated on this task was found after only 1 week of intensive language training, composed of 14 h of formal classes in Scottish Gaelic. Participants were also offered Gaelic entertainment in the evenings including concerts, films and conversation circles. In addition, it was found that those individuals who continued practicing Gaelic for at least 5 h per week following the cessation of the course retained their improvement at the 9 month follow-up. Finally, and perhaps most importantly, improved attentional switching was observed across all age groups, ranging from 18 to 78 years of age, indicating that just 1 week of foreign language training can provide some cognitive benefit even for older learners. In contrast, Ramos et al. (2017) did not observe an improvement in non-verbal task switching ability in older Spanish monolinguals who participated in 8 months of Basque language classes for 5.5 h per week. However, it is not clear why these authors elected to examine task switching as an outcome measure, rather than for example inhibitory control, which would be expected to show some training-related changes following several months of language learning. The domain-general brain circuitry that subserves task switching will not necessarily be affected by language learning *per se*, but rather will depend critically on a bilingual’s pattern of language use. For instance, constant switching between languages (as in the case of codeswitching), or the need to constantly monitor the environment for both languages would be expected to yield improvements in task switching more broadly (Green and Abutalebi, 2013). There are three key differences between the studies conducted by Bak et al. (2016) and Ramos et al. (2017) that may give way to differing outcomes in terms of task switching improvement. The first is the intensity of the initial training. Participants in the Ramos et al. (2017) study had less intense training in the initial week. That is, they participated in three sessions totalling 5.5 h. In comparison, the participants in the Bak et al. (2016) study completed approximately 14 h of language classes, and additional Gaelic language activities. Beyond the initial week, participants in both studies completed at least 5 h of practice in their respective languages. The second difference is that each study measured switching with a different task. Ramos et al. (2017) used the Color-Shape Task, a task commonly used to measure shifting between mental sets, whereas Bak et al. (2016) used the Elevator Task with Reversal, a measure of attentional switching from the Test of Everyday Attention. This can be problematic as these two tasks stem from different theoretical perspectives (the former from working memory, and the latter from attention research) it is not known whether these two tasks measure comparable constructs (see Mackie et al., 2013 for a review on defining cognitive control and attentional functions). The third difference between these studies that may give way to improvement differences, is the context of subsequent language use. While participants in the Ramos et al. (2017) study continued formal classes for 5.5 h per week, those in the Bak et al. (2016) no longer continued their intensive language training. However, it was only those that continued to use Gaelic for at least 5 h per week that improved from their baseline switching performance. Given that language switching is expected to provide improvements to switching more broadly, it is likely that formal classes such as those used in the Ramos et al. (2017) study provide less opportunity for codeswitching, compared to the Gaelic learners who were practicing Gaelic in their everyday lives.

**Table 2:** Summary of studies investigating language learning benefits in older adults.

Study	Sample	Training type	Training duration	Results
Bak et al., 2016	**33 language learners:** intensive Scottish Gaelic course. **16 active controls:** Students of non-language courses. **18 passive controls:** Recruited from Psychology volunteer pool.	Face-to-face intensive course.	14 h over a 1 week period.	Language learners showed advantage for attentional switching using the Elevator Task with Reversal at end of course. Improved switching maintained at 9 months follow-up for those practicing Gaelic for at least 5 h/week after the cessation of the course for all age groups (18–78 years).
Ramos et al., 2017	**26 language learners:** Spanish monolinguals learning Basque. Aged between 60 and 80 (*M*_age_ = 67.42). **17 passive controls:** Spanish monolinguals. Aged between 60 and 78 (*M*_age_ = 69.18).	Face-to-face, small group lessons (maximum 10 participants).	5.5 h (3 days) per week for 8 months.	No decrease in switching cost using the Color-Shape task.
Ware et al., 2017	**14 language learners:** French speaking older adults (*M*_age_ = 75 years) with varying levels of English language knowledge.	Face-to-face group lessons with integrated technology (laptop/tablet).	2 h per week for 4 months. Participants were encouraged to practice using their laptops/tablets outside of sessions.	No difference in cognitive level, loneliness or social isolation. Demonstrated that a technology-based language learning intervention of this duration and intensity is viable for use with this population.

Finally, a recent second language training study aimed to determine whether an English learning program implemented with French-speaking seniors would improve cognition, as well as subjective levels of loneliness and social isolation. Scores on these measures did not improve significantly, perhaps due to the small sample size or short study duration, including the length of the language learning sessions themselves. However, the study did demonstrate that a 2-h per week, technology-based language learning intervention is feasible for seniors to participate in (Ware et al., 2017). Given that Bak et al. (2016) determined that 5 h per week is the minimum level of language use required for cognitive advantages to arise, future research in this area needs to determine whether this also extends to technology-based interventions. Additionally, further research investigating our proposed processing complexity and interference inhibition effects will assist researchers in determining if typological similarity can be used to maximize language training for aging populations. Whether language learning can yield cognitive improvements in older adults, and if so, under what specific conditions, remain open research questions. Answers to these questions are being pursued by research laboratories around the world.

## Language Use in Individuals With Alzheimer’S Disease

One final research question concerns the potential role of language learning in individuals with mild cognitive impairment or Alzheimer’s disease. Studies with Alzheimer’s patients often suffer from design inconsistencies and small sample sizes. However, a picture is starting to emerge regarding bilingual language use in Alzheimer’s disease. Bilinguals diagnosed with Alzheimer’s disease may exhibit cognitive impairment and lapses in attention, decreased language control ability and increased unwanted code-switching (Friedland and Miller, 1999). Bilingual individuals with Alzheimer’s disease show linguistic decrements in both their dominant and non-dominant languages (Stilwell et al., 2016). English dominant bilinguals with Alzheimer’s disease were more likely to name pictures in the non-dominant language than controls; and Spanish-dominant bilinguals with Alzheimer’s disease were equally likely to name pictures in their non-dominant language than controls (Gollan et al., 2011). A case study of two bilingual patients presented early symptoms of dementia after regressing to their primary language (McMurtray et al., 2009). Bilinguals with mild to moderate dementia had impaired retrieval of their first language (Frisian) and L2 (Dutch) naming ability, with a significant effect of age of acquisition. Earlier acquired words were better preserved and retrieved. Qualitatively, inappropriate code switching occurred within the Frisian test setting (Veenstra et al., 2014). These studies provide a glimpse of the effects of Alzheimer’s disease on bilingual language use. Whether language training benefits Alzheimer’s disease patients warrants future investigation.

## Conclusion

In this review, we have outlined the benefits of bilingualism on executive functioning and how this may increase cognitive reserve in older adults. Additionally, we have discussed how foreign language learning programs may potentially promote healthy aging and protect against cognitive decline including Alzheimer’s disease, as a result of the overlap between the brain networks involved in language learning and those that decline in older age. It is proposed that future research in this area should aim to uncover the mechanisms responsible for language learning related brain advantages, and determine how language learning can be optimized to reap the maximum cognitive gains. Specifically, to achieve these aims, future research should determine the role that language typology plays in promoting healthy cognitive aging by systematically manipulating typological similarity in foreign language learning studies. In doing so, language learning programs can be customized to provide maximal cognitive advantage in line with either the processing complexity or interference inhibition effects. We also suggest that more rigorous investigation in this field could be achieved by measuring cognitive abilities prior to, as well as post language learning. Further, we have discussed the potential advantages of bilingualism vs. multilingualism, and suggest that studies that compare cognitive advantages of language learning between monolinguals learning a second language, and bilinguals learning a third, could reveal whether learning additional languages provides an additive effect. Finally, future research needs to determine the optimum language learning conditions that will provide maximum cognitive benefits in older populations. The findings from these lines of research would provide convincing evidence as to whether language learning might promote healthy cognitive aging in older adulthood and, if so, provide guidelines for how these programs should be developed to provide the greatest cognitive advantage.

## Author Contributions

All authors listed have made a substantial, direct and intellectual contribution to the work, and approved it for publication.

## Conflict of Interest Statement

The authors declare that the research was conducted in the absence of any commercial or financial relationships that could be construed as a potential conflict of interest.
